# Identification of IoT Actors

**DOI:** 10.3390/s21062093

**Published:** 2021-03-17

**Authors:** Suada Hadzovic, Sasa Mrdovic, Milutin Radonjic

**Affiliations:** 1 Faculty of Electrical Engineering, University of Sarajevo, 71000 Sarajevo, Bosnia and Herzegovina; sasa.mrdovic@etf.unsa.ba; 2 Faculty of Electrical Engineering, University of Montenegro, 81000 Podgorica, Montenegro; mico@ucg.ac.me

**Keywords:** Internet of things, IoT actor, data manager, GDPR, computing

## Abstract

The Internet of Things (IoT) is a leading trend with numerous opportunities accompanied by advantages as well as disadvantages. Parallel with IoT development, significant privacy and personal data protection challenges are also growing. In this regard, the General Data Protection Regulation (GDPR) is often considered the world’s strongest set of data protection rules and has proven to be a catalyst for many countries around the world. The concepts and interaction of the data controller, the joint controllers, and the data processor play a key role in the implementation of the GDPR. Therefore, clarifying the blurred IoT actors’ relationships to determine corresponding responsibilities is necessary. Given the IoT transformation reflected in shifting computing power from cloud to the edge, in this research we have considered how these computing paradigms are affecting IoT actors. In this regard, we have introduced identification of IoT actors according to a new five-computing layer IoT model based on the cloud, fog, edge, mist, and dew computing. Our conclusion is that identifying IoT actors in the light of the corresponding IoT data manager roles could be useful in determining the responsibilities of IoT actors for their compliance with data protection and privacy rules.

## 1. Introduction

The Internet of Things (IoT) already occupies a significant area, and its perspective is practically unlimited. According to Cisco, there will be 29.3 billion networked devices by 2023 [1]. 

The IoT vision is still evolving as an enabling technology because the IoT keeps developing and new IoT applications are being proposed. Consequently, there is no common IoT definition [2]. In this regard, there is an open call for the contribution of knowledge and perception of the ever-changing definition of the IoT [3].

Because standardization is a process that accompanies the production of new IoT platforms, sensors, and actuators, it has been carried out from the very beginning of the application of this technology. The high complexity of the IoT ecosystem encompasses a wide spectrum of solutions and standards, which is clear from the fact that, in 2016, there were more than 900 IoT-related standards [4].

At the beginning of concept development, typical examples of IoT applications were mostly related to common day to day objects and processes. However, intensive growth of IoT applications moved the focus of implementation towards industrial automation, smart cities, public safety, medical and healthcare systems, and many others. In such circumstances, proper regulation in the IoT domain becomes very important.

To enable synergies for new business models and to reduce barriers, IoT stakeholders must work together and address issues such as interoperability, privacy, and security, and many others, while policymakers need to understand these complex relationships and clearly identify IoT actors and their responsibilities. 

In this regard, as a starting point, we considered some of the most relevant recommendations for the IoT, developed by the International Telecommunication Union (ITU). Accordingly, we analysed the IoT ecosystem and business models given in the ITU Recommendations ITU T Y.4000 [5], ITU T Y.4100 [6], and ITU T Y.4114 [7]. 

To get a complete picture, we have conducted the mappings between the IoT actors and business roles that were given in the selected ITU recommendations and from various other perspectives (regulatory, product development and technology consultancy companies, industry organizations, and other).

The scope of the IoT involves different sectors and, consequently, various authorities are present, such as electronic communications regulatory authorities; data authorities; regulators and ministries for energy, health, air security, traffic, transport; and others. Consequently, more open, collaborative, and cross-sectoral regulation is needed. IoT policies and regulations are still under development and it is important to encourage a coordinated regulatory approach that includes all sectors. This concept of “collaborative regulation” and “fifth-generation regulation”, originally developed by the ITU, is the only viable solution in this Data Age, where IoT development enables the generation of a huge amount of data by various data sources [8]. 

Given the wide range of regulatory challenges, we focused our work on regulatory challenges with an emphasis on the data protection and privacy aspects. In this regard, in the IoT legislative landscape of EU, the General Data Protection Regulation (GDPR) [9] and draft ePrivacy Regulation [10] are standing as trust drivers, and correct identification of roles such as data controller, joint controllers, or data processor and allocation of corresponding responsibilities would be extremely demanding. The situation in the case of IoT is even more difficult if we keep in mind that IoT is characterized by joint controllers who have complex and different shares of corresponding responsibilities. 

A review of existing IoT architectures in Alshohoumi et al. [11] identified sixteen different IoT architectures that were developed during the period from 2008 to 2018, emphasizing the gradual evolution of IoT architecture across the years. It is shown that, from the layered architecture perspective, IoT extends from an early three-layer architecture model to the eight-layer architecture model. Based on analysis of seven IoT architectures, authors in Lynn et al. [12] summarized that key features in IoT reference architectures include data management, security and privacy, analytics, data visualization and user interface, supported computing paradigms, scalability, and interoperability.

Different standardization groups work continuously on reference models for IoT architectures. Acknowledging the existence of many IoT architectures, we attempt to find a correlation between the basic model of the network for the IoT presented in Recommendation ITU-T Y.4113 [13], Cisco IoT simplified architecture [14], the conceptual model of fog and mist computing for IoT given in the publication by National Institute of Standards and Technology (NIST) [15], the architectural reference models of devices for IoT applications in Recommendation ITU-T Y.4460 [16], and the IoT value chain as a useful tool for regulatory authorities.

There is existing related study into the identification of various stakeholders in the IoT value chain model on Smart Cities, where authors proposed taxonomy that categorizes and lists the relevant technology and regulatory characteristics of Smart City services [17]. Regarding computing paradigms, the authors in Yousefpour et al. [18] provide an extensive tutorial on fog computing and related computing paradigms and identify relevant operators and computing hardware locations. The authors in Ray [19] and Šojat and Skala [20] give an introduction to dew computing and identify dew computing locations.

Different from these studies, the contribution of this paper is three-fold: (1) We provide a detailed mapping between the IoT actors identified from various perspectives. (2) As part of the identification of IoT components and IoT actors, we identify new data manager roles related to dew, mist, edge, fog, and cloud computing. (3) We have compiled it into a new five-computing layer IoT model based on the cloud, fog, edge, mist, and dew computing, including identified IoT actors and additional roles according to the different computing paradigms and the GDPR. This IoT model can serve as a valuable support in clarifying IoT components, IoT actors, and corresponding GDPR roles.

The paper is organized as follows: Section 2 gives a short overview of GDPR and draft ePrivacy Regulation, with a focus on identification of GDPR actors. Section 3 presents a detailed description of the IoT components. Section 4 is focused on computing paradigms in IoT and the most common fog, edge, mist, and dew computing hardware locations. Next, in Section 5, mappings are provided between the IoT actors identified in selected ITU recommendations and those given from various perspectives (regulatory, product development and technology consultancy companies, industry organizations, and others). Section 6 introduces the proposed IoT model with the identified IoT actors, relevant GDPR actors, and the new data manager roles related to dew, mist, edge, fog, and cloud computing. Section 7 gives a short overview of current regulatory status and the need for a collaborative regulatory approach. Section 8 gives a short overview of data brokers, gatekeepers, and other actors in light of recent legislatives. In Section 9, conclusions are drawn.

## 2. The Data Protection and Privacy Legislative Landscape in the EU

The IoT is an important part of the current Data Age reality where, parallel to IoT development, significant privacy and personal data protection challenges are also growing. The GDPR and draft ePrivacy Regulation in the EU are dealing with these issues.

### 2.1. GDPR and Implementation Guidelines

Core activities regarding data are creation, collection, storage, aggregation and organization, processing and analysis, marketing and distribution, and use. Support activities regarding data are data laws, regulations, and policies; data security and privacy-related service; ICT (Information and Communication Technology) connectivity and infrastructure services; and data skills enhancement services. Data laws, regulations, and policies can address data and data rights ownership, data classification and metadata, data protection and security, data privacy, data transparency and consent, and data commercialization [21].

GDPR entered into force in 2016, and all organizations who target or collect data related to people in the EU were required to be compliant with GDPR as of May 25, 2018 [9]. Data protection by design and by default is mandated; consequently, data protection should be considered both at the stage of the determination of the means of the processing as well as at the time of the actual processing (Article 25). The concept and interaction of data controller and data processor are central.

Given the existing complexity in defining corresponding roles, relevant guidelines have been published to clarify the roles of the controller, joint controller, and processor, and the distribution of responsibilities among them.

The European Data Body Supervisor issued guidelines to the EU institutions regarding their role in the processing of personal data on 7 November 2019. Although the guideline is limited to EU institutions, it can be very useful for all business in determining their role as controller, joint controller, or processor under the GDPR. It is clarified that an entity does not need to have access to personal data to be a controller as long as it has an influence on processing, determines the purposes and means of processing, or receives the anonymous statistics based on personal data collected and processed by another entity. The duties of controllers and processors are explained in Annexes 2 and 3, while the flowchart is given in Annex 1 for a situation in which the distribution of roles of processors and controllers has not been determined by a legal act [22].

The European Data Protection Board has issued guidelines on controller and processor concepts in the GDPR for open public consultations from 2 September 2020 to 19 October 2020. The guidelines clarify that joint control can be based on a joint decision of two or more multiple entities or through a convergent decision of two or more entities [23].

Proposed guidelines for meeting the GDPR principles is also given in European Telecommunications Standards Institute (ETSI) Technical Report ETSI TR 103 591 (2019-10) [24].

Under the GDPR, supervisory authorities and the EU Commission are allowed to issue standard clauses to be included in the contract between processors and controllers, providing a way to ensure that the contract complies with the GDPR [25].

The first European Commission evaluation and review of the GDPR, published on 24 June 2020, emphasizes the importance of clarifying how to apply proven principles to specific technologies such as IoT, artificial intelligence, blockchain, and facial recognition. The implementation of the GDPR, as opposed to large digital companies and integrated companies, has been recognized as an essential element for the protection of individuals. The right to data portability, which enables individuals to switch between different service providers, is considered one of the Commission’s priorities, particularly with the increasing use of the IoT [26].

A short overview of GDPR actors is given in Table 1.

### 2.2. Draft ePrivacy Regulation

In January 2017, the European Commission published a Proposal of Regulation on Privacy in Electronic Communications (draft ePrivacy). On January 5, 2021, the Council of the European Union released the 14th draft version of the ePrivacy Regulation. [10]. It is broader than the GDPR because it applies not only to the processing of personal data but also to the processing of any electronic communications data and other data collected from the end user’s device. The goal is to safeguard the integrity of end user devices and the privacy and confidentiality of their communications.

In the proposed text, Recital 12 states that, to ensure full protection of the rights to privacy and confidentiality of communications and the promotion of a reliable and secure IoT, the proposed regulation should apply to machine-to-machine communications transmission.

Recital 17 states that electronic communication networks and service providers should be permitted to process electronic communications metadata after obtaining the consent of the end user or, where necessary, to provide an electronic communications service under an end user contract, and these where necessary to protect an interest that is essential for the lives of end-users.

Proposed taxonomy for personal data in the context of the telecommunication sector in [27] depicts what kind of data is accessible by different actors. For example, Fixed Network Operators, Mobile Network Operators, and Mobile Virtual Network Operators have access to content data in clear text but cannot use it. Over to the top (OTT) service providers can access the data for the service they provide. Device manufacturers/Operating system providers can access data before it leaves the device.

## 3. IoT Components

The IoT consists of various components or building blocks, and there are a variety of approaches to identify IoT components such as those included in the basic model of the network for the IoT, identified in Recommendation ITU-T Y.4113, which consists of Device, IoT area network, Gateway, Access Network, Core network, IoT Platform, and IoT application server [13]. The IoT infrastructure identified in the paper [28] consists of IoT Devices, IoT Platform, Fog nodes, Cloud nodes, and IoT Applications.

To develop the effective legislation further, regulatory authorities need to have a better understanding of IoT building components, who the IoT actors are, what are their relationships, and how IoT building components add value to the IoT solution and for the end-user. In this regard, the value chain model could be an example of a useful analytical tool for regulators. At the same time, the IoT value chain presents a challenge as IoT is evolving, and it involves various IoT actors and building components with dynamic and unclear relationships between them. Some of the components of the IoT value chain are: Device, Connectivity, as identified in Mackenzie and Rebbeck [29,30] and Paradis [31], Applications, identified in Mackenzie and Rebbeck [29,30], IoT platform enablement, identified in [30], System Integration, identified in Mackenzie and Rebbeck [29,30], Service enablement, identified in Paradis [31], Service Provision, identified in [30], and Customer, identified in Paradis [31].

From the above examples of identification of IoT components, it can be concluded that the identified IoT components sometimes have a certain degree of overlap. In the following, we would like to point out some IoT components that we believe would be useful for regulators to understand the relevant processes and relationships among IoT actors, noting that the list of possible IoT components is not exhaustive.

### 3.1. Thing

As for the IoT, the ITU has recognized the Thing as an object of the physical world or the information world, capable of being identified and integrated into communication networks [5]. Sometimes, Thing is integrated into a smart device itself, or Thing stands alone and a separate product is connected, making it a smart device. Although the ITU basic network [13] does not present Thing alone, our point of view is that Thing needs to be visible as a building block.

### 3.2. Device

The IoT is full of new terms, such as the Mote, which stands for Remote, where Motes make up a significant portion of the IoT [32]. The ITU has defined Mote as a miniature computing device equipped with sensors and signal transceivers operating in a specific radio band and used to transmit sensed data [33].

While acknowledging the existence of various terms, our focus is on Device as an elementary IoT building block. Device is identified by the ITU as a piece of equipment with the mandatory capabilities of communication and optional capabilities of sensing, actuation, data capture, data storage, and data processing [5].

Regarding actuation and sensing capabilities, an actuator performs physical actions caused by an input signal and a sensor senses chemical compounds or monitors environmental conditions and sends an electronic signal proportional to the sensed value. The Global System for Mobile Communications Association (GSMA) considered generalized IoT Device Architecture as a combination of IoT Device Host and IoT Device where IoT Device consists of the IoT Device Application and Communications Module (consisting of Communications Module Firmware, Radio Baseband Chipset, and Universal Integrated Circuit Card) [34]. The IoT Device is a combination of software and hardware. IoT device hardware typically consists of thing and modules for data acquisition, data processing, and communication, while IoT device software consists of operating systems and device applications [35].

Our focus is on the ITU classification of devices regarding processing capabilities [16], as follows:

- Devices with no processing capabilities (a low-cost device with no microcontrollers and without processing capabilities);
- Devices with low processing capabilities (a low-cost device with very limited microcontrollers and processing capabilities, used only for reading or writing data from/to sensors/actuators and sending or receiving those data);
- Devices with high processing capabilities (devices with processing capabilities for making decisions, running algorithms, and directly coordinating with other devices).

If a correlation of the processing and communications is pursued, bearing in mind that the combination of high processing and low connectivity is not usual, it is possible to list three types of device [16]:

- Device with low processing and low connectivity (LPLC);
- Device with low processing and high connectivity (LPHC);
- Device with high processing and high connectivity (HPHC).

### 3.3. Gateway

A Gateway [36] interconnects devices with communication networks and performs the necessary translation between the protocols used in the communication networks and those used by devices. Acknowledging that some IoT solutions do not require a gateway, the gateway must be identified as a basic IoT component.

### 3.4. Connectivity Network

Many connectivity technologies can be used in IoT. They range from wired to wireless technologies as a trade-off between bandwidth, range, and power consumption. Consequently, one of the classifications [37] may be as follows:

- High range, high power consumption, and high bandwidth (Cellular, Satellite);
- High range, low power consumption, and low bandwidth (LPWANs);
- Low range, low power consumption, and high bandwidth (Ethernet, Bluetooth, Wi-Fi).

Besides traditional connectivity networks, other networks also appear. For example, sensor control networks are used increasingly for a variety of applications. The ITU has defined a sensor control network as a sensor network consisting of Motes intended to control one or more actuators [33]. The IoT area network, defined by the ITU, is a network of IoT devices and gateways interconnected through local connections [13].

### 3.5. IoT Platforms

The ITU has defined the IoT platform as a technical infrastructure that provides the integration of generic and specific capabilities. These capabilities, in conjunction with the capabilities of the core network, may be exposed to IoT application servers. The core network enables communication functionalities for supporting the data transfer to devices and gateways via the access network. Some of those functionalities can be used by service providers [5].

There is no standard IoT platform configuration, and there are a variety of IoT platforms. Currently, stated in IoT Analytics IoT Platforms Company Landscape 2020, there are officially 620 IoT Platform companies on the open market [38]. The possible classification of IoT platforms [39] fall into five main types:

- Connectivity platforms;
- Device management platforms;
- Cloud platforms;
- Application enablement platforms;
- Advanced analytics platforms.

The IoT Platform market is concentrated around a few well-known key providers because the market share of the top 10 platforms is 58%. The focus area is primarily on Manufacturing/Industrial use, Energy, Mobility, Smart Cities, Health, Supply Chain, etc. There are multiple benefits for IoT Platform vendors to create open ecosystems and cross-vertical and cross-value chain collaborations through the IoT platform itself or by creating formal alliances and partnerships with vendors at multiple levels of the value chain. Some of the benefits are cost reduction, enhancing security, shortening time to get into the market, the speed of innovation increases, etc. [40].

In ITU Recommendation ITU-T Y.4208 has identified a new IoT component, the edge platform, which is usually a kind of cloud platform. It is about transferring some IoT capabilities from IoT application server and IoT platform to the edge platform, aiming to support edge computing. The edge platform is situated between the access network and the core network [41].

### 3.6. IoT Application Server

According to ITU definitions, the IoT application server runs applications and communicates with devices, gateways, and the IoT platform via the core network (or directly, in the case of communicating with the IoT platform) directly to deliver application services [13].

### 3.7. IoT Application

Application is a structured set of capabilities that provide value-added functionality supported by one or more services [42]. IoT application can be referred to as application provided by an IoT application provider.

### 3.8. IoT Service

Service is a structure set of capabilities for applications support [42]. However, there is still no clear definition of the IoT service given that IoT services are constantly evolving and taking different forms [43]. The IoT service can be referred to as a service provided by an IoT service provider.

### 3.9. IoT User

The “IoT user” actor is an IoT actor that uses all possible services related with things, such as monitoring, location tracking, and service discovery, defined by ITU Recommendation ITU-T Y.4100 [6]. IoT user is defined by the Body of the European Regulators of Electronic Communications (BEREC) as the purchaser of an IoT service who incorporates the IoT service as one component in his own products and/or services [43].

### 3.10. End User

End user is defined by the ITU as the actual user of the products or services offered by the enterprise. The end user consumes the product or service [44].

### 3.11. IoT Data Protection and Privacy

Data protection and privacy must be ensured in the IoT. Designation of a data protection officer is needed because the IoT fulfils the requirements of Article 37, paragraph 1, GDPR, in such a way that processing operations , by virtue of their nature, scope, and/or their purposes, require regular and systematic monitoring of data subjects on a large scale [9].

### 3.12. IoT Security

Security must be ensured for data in use (device level), idle data (stored data), and data in motion (data transported across a network). Some of the possible consequences of inadequate IoT security could be loss of privacy, danger to health and safety, theft of data from the system or theft of material items, danger of reputation, loss of productivity, and noncompliance with laws or regulations, etc. Therefore, IoT security is a central issue and must be implemented along with the entire IoT system, which means at the device level, in the network, cloud, etc.

IoT devices are going to be more vulnerable (for example, low-cost nodes with low budget for security, low compute power for encryption) and easily accessible to attackers (for example, smart light bulbs, smart thermostats) than traditional IT systems. Additionally, the exponential growth of IoT connected devices means a larger area for attackers. Consequently, IoT security is more challenging than cybersecurity. It starts with cybersecurity, and further security measures are needed [45].

It is recommended that device manufacturers perform certain cybersecurity activities to provide the necessary cybersecurity functionality of IoT devices and to provide related information to customers. In this regard, the comprehensive guidelines for IoT device manufacturers, issued by the National Institute of Standards and Technology (NIST), classified those specific activities that have primarily a pre-market impact and activities with primarily a post-market impact [46] while providing, as a starting point, a set of IoT device cybersecurity capabilities for manufacturers [47].

There are many available IoT security certification schemes; one example is the Eurosmart IoT Security Certification Scheme for the IoT Device, defined by the Cybersecurity Act, with a focus on the Substantial security assurance level. The goal is ensuring that certified IoT devices comply with specified requirements throughout their life cycle [48].

The ETSI released ETSI EN 303 645 in June 2020, which is a consumer IoT Security standard specifying 13 provisions for the security of Internet-connected consumer devices and their associated services [49]

The Cyber Security Act established the EU wide cybersecurity framework for ICT products, services, and processes on 27 June 2019. The European Commission will be required to conduct periodic assessment if specific cybersecurity requirements become mandatory for certain ICT processes, services, and products. In this regard, from a consumer perspective, the ENISA (The European Union Agency for Cybersecurity) Advisory Group’s working group on cybersecurity calls for mandatory certification schemes for certain ICT products, services, and processes instead of the current EU-wide voluntary certification scheme. Responsibility for implementation and supervision of the schemes is assigned to National cybersecurity certification authorities [50].

There are different certification requirements for IoT devices, which can be classified as follows [34]:

- Regulatory certification (FCC, EC);
- Industry Certification;
- Telecoms: the two main Telecoms Industry certification schemes are the Global Certification Forum (GCF) and the PCS Type Certification Review Board (PTCRB);
- Operator Certification (Deutsche Telecom, Verizon, AT&T).

## 4. Computing in the IoT Ecosystem

General issues of the network for the IoT are identified in ITU Recommendation ITU Y. 4113 [13], as follows:

- Packet loss and higher latency;
- Unreliability of short-range radio communications in the IoT area network;
- Network overload due to large amount of traffic to be processed.

The largest amount of computing capability is tied to cloud computing. However, cloud computing poses a substantial problem in supporting time-critical and location-aware IoT applications because it relies on remote and centralized resource provision. As more devices are expected to be connected to the Internet, problems with high latency, poor security, poor reliability, high network bandwidth, storage costs, communication power, and more are expected to grow, as discussed in Silva et al. [51] and Jiang et al. [52].

The authors in Li and Wang [53] point out the shortcomings of cloud computing in solving possible problems encountered with IoT and explain the possibility for solving these problems and introduce fog computing. Recognizing that cloud and fog roles are complementary, the authors in Bonomi et al. [54] claim that there is a fruitful interplay between the cloud and the fog, especially when it comes to analytics and data management.

### 4.1. Computing Models

The term fog computing, introduced by Cisco in 2014, is tied to the decentralization of computing infrastructure. Cisco’s simplified IoT architecture consists of basic building blocks with security across the entire architecture and data management aligned with each layer of the core functional stack. The three IoT data management layers are [14]:

- The edge layer, where data management takes place within the sensors themselves;
- The fog layer, where data management takes place within the gateways and transit network;
- The cloud layer, where data management takes place within the cloud data centre.

On March 2018, NIST released a publication presenting a fog and mist computing conceptual model, together with their relationships to cloud computing models. In the model, the fog node is presented as a physical component, such as gateway, server, router, switch etc., or a virtual component, such as a virtual machine, etc. Mist nodes are located at the edge of the network, directly within the network fabric where they use microcontrollers and microcomputers. It is underlined that edge computing is considered as a network layer that envelops end devices and their users [15].

On the other hand, Cisco considers fog computing, micro data centres, multi-access edge computing, cloudlets, and emergency response units as five types of edge computing [55].

As an extension of the existing client-server architecture, a new four-tier architecture has been proposed in Ray [19]. This architecture consists of a cloud, fog, edge, and dew layer and makes it easier for the user to access web data from any sources (edge, fog, or cloud) through minimal or no Internet access.

### 4.2. Fog, Edge, Mist, and Dew Hardware Computing Locations

The authors in [18] provide an extensive tutorial on fog computing and related computing paradigms. The observed computing paradigms included cloud computing, cloudlet computing, mobile computing, edge computing, mist computing, and other similar computing paradigms. Although there are many computing paradigms, it is emphasized that some paradigms are a subset of others; for example, mobile computing is a sub-set of mist computing and edge computing is a sub-set of fog computing, etc. Though not identified in [18], there is another type of computing, namely, dew computing, which aims to enable content when there is no Internet connection.

Many researchers are trying to figure out where all these computing nodes are located. Dew computing uses mostly on-premises computers, while fog mainly includes routers and sensors in the IoT [56]. Dew is identified by the authors in Ray [19] as a server inside the user’s PC. According to Šojat and Skala [20], Dew computing happens in information processing devices located, for example, in refrigerators, car motors, traffic-controls, lights, theatres, and industries. In addition, it is emphasized that the benefits of integrating dew devices into the cloud-fog-dew hierarchy are very significant.

Mist computing puts computing power at the far edge of the network, and usually consists of microchips or microcontrollers built into the device [57]. Mist computing uses microcomputers and microcontrollers for sending data to fog nodes and the cloud if needed. In the mist layer, sensor data pre-processing is performed, and only the essential data is sent to the gateway, server, or router, which saves bandwidth and battery power [58].

Edge computing can be used to process data in near real-time by processing data closer to the edge, directly on devices that have attached sensors or gateway devices that are close to the sensors. Edge computing is less scalable compared to fog computing and supports low interoperability, making some IoT devices incompatible with some operating systems and cloud service.

Authors in the paper [59] discussed fog computing, mobile edge computing, and cloudlet computing in detail, together with comparisons of their features. In this regard, fog computing node devices are identified as routers, access points, switches, and gateways while, for mobile edge computing, node devices are identified as servers running in base stations and cloudlets running a virtual machine. A lack of standardization and different interpretations by different consumers was also emphasized.

Fog computing is characterized by placing computing capability in a connection between device sensors and a cloud server, usually in a device that acts as a gateway, connecting the sensors and managing the connection to the cloud. Computing decentralization is achieved by processing data in a fog node, and it can be any device capable of computing, data storage, and network connectivity. Fog computing can reduce latency and process larger amounts of data compared to edge computing due to its ability to process requests in real-time [60]. Fog computing and edge computing differ in intelligence location identification and power computation. In the case of edge computing, processing power and intelligence are placed in devices such as built-in automation controllers while, in the case of fog computing, intelligence is placed in the local area network while edge devices and the gateways along local area networks are used for power processing [61]. The fog layer supports local data storage, data filtering, compression, merging, and intermediate analytics to save backbone bandwidth, reduce the cloud load, and improve system performance [58]. The fog node is identified as a mini cloud, located at the edge of the network, where the most common fog locations are in high-performance devices, such as smart gateways or routers [60].

The authors in [18] identified hardware locations for cloud computing as being large data centres; hardware locations for fog computing are devices with virtualization capacity as servers, routers, switches, access points; hardware locations for edge computing are edge devices with computing capability; and hardware locations for mist computing are IoT devices (e.g., sensors, cell phones, home appliance devices). Additionally, cloud service providers are identified as operators for cloud computing, users and cloud service providers as operators for fog computing, network infrastructure providers or local business as operators for edge computing, and self-organized or local business as operators for mist computing.

In the situation of there being no consensus on the distances among more computing paradigms, it can be concluded from the above computing location identifications examples that sometimes computing hardware locations have some degree of overlap. Based on the analysis, our approach is summarized in Table 2.

As all these computing technologies have some advantages and disadvantages, the use of all these types of computing will be key to ensuring the ability of applications and systems to scale alongside a growing network of devices [62].

According to the vision expressed in Roberts [63], the biggest IoT transformation will be in shifting power in the network from the centre to the edge. Therefore, the IoT will allow devices to directly communicate with each other rather than communicate through cloud-based management servers or central hubs.

## 5. IoT Actors

The IoT ecosystem consist of multiple coexisting and competing platforms and products, along with a variety of business players interacting with each other. In this regard, we found that certain models introduced by the ITU could be a starting point suitable for research and further adaptation. An Informative Appendix I of Recommendation ITU-T Y.4000 [5] presents an example of identified business roles in the IoT ecosystem and their relationships. As this example does not represent all possible relevant roles in IoT business deployments, we intend to extend it to include the impact of new computing paradigms. We also consider Recommendation ITU-T Y. 4100 [6] because it provides common IoT requirements based on the general use cases of the IoT and IoT actors. In light of the expectation that the number of connected things will be so enormous that the IoT data will constitute a predominant part of the data carried by networks, Recommendation ITU-T Y.4114 [7] and presented key possible mappings from IoT business roles [5] to the IoT data roles are also taken into account in this research.

Business roles identified in the Informative Appendix of Recommendation ITU-T Y.4000 [5] are as follows:

- The device provider provides devices to the network provider and application provider;
- The network provider performs access and integration of other provider resources, provides IoT capabilities and their support and management of their infrastructure, and provides network capabilities and resources to different providers;
- The platform provider provides capabilities to application providers, such as data storage, data processing, device management, integration capabilities, and open interfaces;
- The application provider provides IoT applications to application customers while using capabilities or resources of the network provider, device provider, and platform provider;
- The application customer is the user of the IoT applications provided by the applications provider.

Applicable mappings between IoT actors described in ITU Recommendations [6] and [7] with IoT business roles described in Appendix I of [5] are presented in Table 3.

Analysing these mappings between the IoT actors and business roles in selected ITU recommendations, and taking into account the definitions for ‘Data Manager’ given in ITU-T Y.4100 [6], ‘Device Provider’ and ‘Application Provider’ given in ITU-T Y.4000 [5], identified as Data manager corresponding business roles, our view is that Data Manager as an IoT actor needs to be more granulated. With more granulation, the overall data flow and corresponding responsibilities become more understandable and clearer.

Data Manager actor corresponds to Device Provider when provided devices that involve some data management functionalities [6]. Depending on the provided device processing capabilities, the corresponding Data Manager actor needs to be granulated as Dew Data Manager, Mist Data Manager, Edge Data Manager, Fog Data Manager, and Cloud Data Manager.

Data Manager actor corresponds to Application Provider when the provided applications involve some data management functionalities [6]. As the Application Provider uses the resources or capabilities of the device provider, network provider, and platform provider, the corresponding Data Manager actor needs to be granulated as Network Data Manager, Platform Data Manager, and Application Data Manager.

A variety of device options and use cases, combined with a variety of IoT applications, makes the IoT value chain a complicated ecosystem that can have a countless number of partnerships between the participants.

Additionally, designation, manufacturing, and distributing of IoT devices can be done with incompatible standards in different jurisdictions. The situation is the same with IoT actors, i.e., many of them are outside the jurisdiction in which the IoT service is delivered.

Therefore, we will consider additional perspectives (regulatory, product development and technology consultancy companies, industry organizations, and others) on how the market players in the IoT value chain are understood, and these are summarised in Table 4.

## 6. IoT Model

As the focus in the IoT ecosystem is on data, monitoring the flow of data is complex. Multiple roles of IoT actors are possible, and some IoT actor could be IoT data market stakeholder at the same time or have relations with IoT data market stakeholders. Identifying all these IoT actors and clarifying their roles and responsibilities is of great importance regarding various aspects.

**Figure 1:**
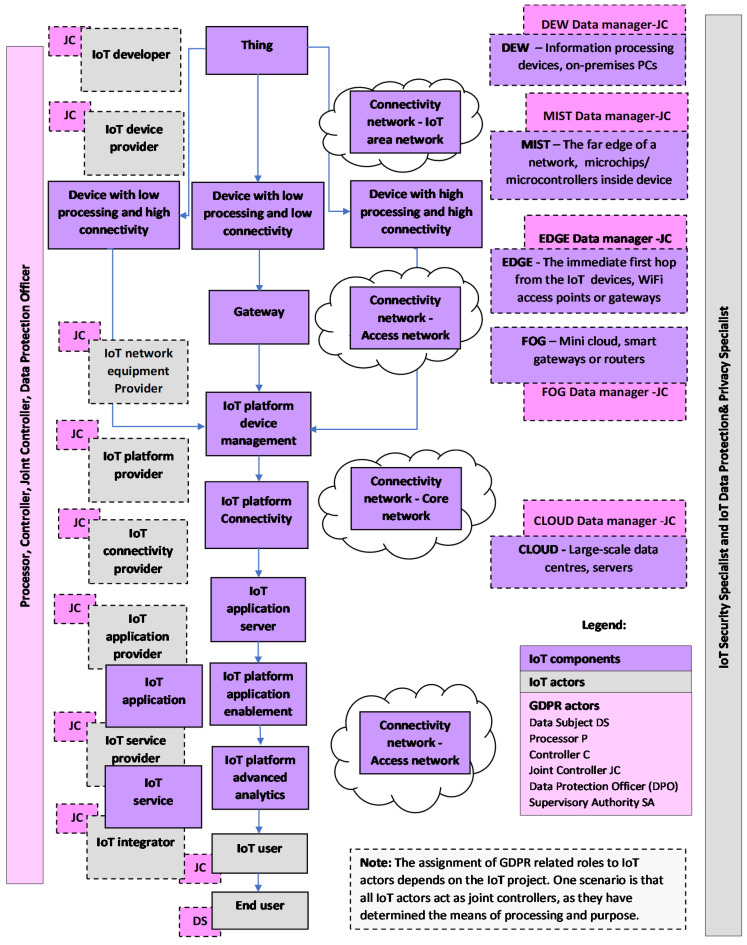
IoT Model.

As the IoT deals with unlimited heterogeneous connected devices, there is a need for a flexible layered architecture. Keeping in mind the previously presented various perspectives, the diversity of IoT concepts and inconsistencies is evident. Although there is no all-encompassing IoT architecture in place, there are some key components and features that are shared in most IoT deployments. Therefore, the previously presented IoT concepts can be adapted to a new IoT model, presented in Figure 1.

### 6.1. Methodology for Identifying IoT Components and IoT Actors

We used the basic model of the network for the IoT, identified in Recommendation ITU-T Y.4113 as a starting point. This model consists of: Device, IoT area network, Gateway, Access network, Core network, IoT platform, and IoT application server [13].

We used all these components in making a new IoT model, but acknowledging that other networks may appear, we present an IoT area network, Access network and Core network as one Connectivity network. To make it easier to understand, we present all three networks in Figure 1.

We add Thing as an object of the physical world or the information world, capable of being identified and integrated into communication networks (for example, a human being is a Thing in the case of remote diagnostics and health monitoring)

Acknowledging the existence of a potentially unlimited number of diverse IoT Platforms, to make it easier to understand, we present the five most common IoT platforms, i.e., Connectivity platforms, Device management platforms, Cloud platforms, Application enablement platforms, and Advanced analytics platforms, in Figure 1.

The connectivity network is combined with IoT network equipment and the IoT Connectivity platform, and it is operated by an IoT Connectivity provider.

Device is identified by the ITU as a piece of equipment with the mandatory capabilities of communication and optional capabilities of sensing, actuation, data capture, data storage, and data processing [5].

The Device is delivered by the IoT Device provider while the IoT Device management platform unifies and simplifies the management of IoT devices, the provisioning of software updates to devices, and offers other functionalities. Network equipment is provided by the IoT network equipment provider.

IoT Security is mandatory in the IoT. Therefore, all related activities aiming to deliver security may be considered as IoT components.

IoT Data Protection and Privacy is mandatory in IoT. Therefore, all related activities aiming to ensure data protection and privacy may be considered as IoT components.

IoT user is the purchaser of an IoT service who incorporates the IoT service as one component in their own product and/or service (It could be a car manufacturer or electricity provider who includes a smart meter in their services).

End user is the actual user of the products (a car owner, user of applications and services).

IoT application can be referred to as the application provided by an IoT application provider, while IoT service can be referred to as the service provided by an IoT service provider.

IoT integrator is the IoT actor who delivers end-to-end solutions.

IoT developer is focusing primarily on the creation of software. 

### 6.2. Methodology for Identifying Relevant GDPR Actors

In the EU, Article 29 Data Protection Working Party issued a specific Opinion 8/2014 on the Recent Developments of the Internet of Things on 16 September 2014, emphasizing that IoT stakeholders should ensure that data at every level is used for purposes known to the user and compatible with the original purpose of the processing. Accurate identification of the involved IoT stakeholders is necessary to qualify their legal status as data controllers who must comply with various obligations. It is stated that most device manufacturers collect and process personal data generated by the device, which qualified them as data controllers. Third-party application developers, unless the data is properly anonymised, must be considered as data controllers. Other third parties may use IoT devices to collect and process information about individuals, so they are also qualified as data controllers. IoT data platforms can also qualify as data controllers for processing activities for which they determine purposes and means, under EU data protection law [72].

In the case of the IoT determining controller or processor roles, this is always dependent on the characteristics of the actual IoT project [73].

Data Protection Officer (DPO) is generally necessary in IoT, because of the large scale of personal data processing. The DPO informs and advises the controller or the processor, monitors compliance with the GDPR, provides advice when required, and acts as the contact point for the supervisory authority. In Figure 1, Data protection and privacy specialist and DPO are separate, but this specialist may be appointed as DPO. DPO guides the company toward GDPR compliance. Large companies have departments to handle data protection and privacy related matters.

Data protection impact assessment (DPIA) is of great importance in the IoT, and where the DPIA indicates a high risk, the controller must consult the supervisory authority.

Supervisory Authority is a public authority in EU Member States, and it is also typically referred to as Data Protection Authority or equivalent.

Lead Supervisory Authority is applicable in the case of multinational companies where the company may choose not to appoint a DPO for each country of operation.

Controller determines the purposes and means of the processing of personal data, while the processor processes personal data on behalf of the controller, according to the Data Processing Agreement signed with the controller.

It is often possible to have joint controllers where every IoT actor determines the purposes and means of the processing of personal data.

For example, an IoT integrator who delivers end-to-end solutions could be only one controller, while other IoT actors are processors who processes personal data on behalf of the IoT integrator according to the Data Processing Agreement signed with the IoT integrator as the controller.

## 7. Current Status and Collaborative Regulatory Approach

Every year, during ITU Global Symposiums for Regulators (GSR), the Best Practice Guidelines are adopted by the global community of ICT regulators. Recent GSR2020 Best Practice Guidelines point out that IoT is one complex issues that is waiting to be addressed. As new issues call for novel approaches, formal regulations should leave enough space for self-regulation and hybrid and collaborative regulatory models [74].

A cross-sectoral IoT nature requires a cross-sectoral regulatory approach for maximizing the IoT benefits while minimizing the IoT risks. As ICT underpins almost every sector of the economy, traditional ICT sector regulations in the silo-style is not viable anymore. This need has been demonstrated in practice based on the analysis of the main barriers to the adoption of smart city IoT projects that were identified while research was being conducted on the assessment of more than 350 projects, which are funding, silos, and politics [75].

The ITU has developed the concepts of “collaborative regulation” and “fifth-generation regulation”, according to the concept of ICT regulation generations. Currently, the status is far from satisfactory from the data on the G5 Benchmark (The Benchmark of Fifth Generation Collaborative Regulation) in the Global ICT Regulatory Outlook 2020, issued by the ITU. The G5 Benchmark covers 80 economies from all regions on the glide path towards collaborative regulations, and it uses 2018–2019 data. It shows that nine countries out of every ten are still regulating the ICT sector as a separate economic sector, while only sixteen countries in total have a holistic and forward-looking regulatory framework. Eight indicators out of a total of twenty-five belong to the assessment of collaboration degree, measured between the ICT regulator and the Competition Authority; the Consumer Protection Commission; the Data Protection Commission; the Spectrum Agency; the Broadcasting Regulator; the Financial Regulator; the Energy Regulator, and the Internet agency [8].

A broader picture of the current regulatory state can be obtained if the ICT Regulatory Tracker is also considered. This tracker, issued by the ITU, shows the evolution of the four generations of ICT regulation whereby, in 2019, only 32.6 % of 193 countries belonged to the fourth generation of ICT regulations [76].

The collaboration was recognized as a cornerstone of success in the annual IESE Cities in Motion index, which examines all aspects of quality of life and sustainability in 181 key global cities. According to the report [77], the best ranking cities fully understand that the challenges are too great to be addressed individually and indicate that collaboration is key for achieving long-term success.

Currently, involved authorities in the data economy are Competition authorities, Data protection authorities, Electronic Communications National Regulatory authorities (NRAs), Cybersecurity authorities, and Governmental offices promoting open data policies/information fairness. Data protection authorities are responsible for the application of the GDPR and, in some cases, the ePrivacy Directive, while NRAs are responsible for the regulation of the telecoms market and application of the ePrivacy Regulations [27].

## 8. Data Brokers, Gatekeepers, and Other Actors

As the number of devices increases, the amount of data collected by these devices also increases. According to the Statista report, the total data volume of connected IoT devices worldwide is projected to reach 79.4 zettabytes by 2025 [78].

Across the IoT, data is created by devices and sent to applications to be sent, consumed, and used. A new/old actor appears—a data broker exploits and sells personal data about individuals to third parties. According to the USA Federal Trade Commission (FTC) report released in May 2014, data brokers are companies that collect consumers’ personal information and resell or share that information along with others. The data broker industry is complex, consisting of collecting consumer data, mostly without their knowledge, combining online and offline data, and analysing data about consumers to make visions of consumers. Commonly, multiple data brokers provide data to each other [79].

Despite the terms and conditions for privacy, as the opt-in-based agreement provided by the data brokers, the data providers (or data sources) still do not know how their data is being processed, delivered, and used. So far, IoT data markets have not been well-formed due to lack of transparency between providers and brokers/consumers [80]. Current IoT data markets are classified into two types of market, as privacy protection markets and privacy valuation markets.

Authors in Oh et al. [81] considered the following four major stakeholders for modelling the IoT data market:

- Data providers;
- Multiple data brokers who collect raw data from various source and sells big data;
- The data service provider who utilize big data from the data brokers;
- Service consumer.

The findings of Wolfie [82] emphasized that data brokers, online platforms, advertising technology providers, and business in industries can now monitor and analyse individuals in various aspects. As a result of the recent technology developments, we are talking about unprecedented new qualities of ubiquitous corporate surveillance with potential danger that could end in a society without privacy. Much of these activities occur in the background and remain blurred to most consumers as well as to policymakers. It is no secret that many companies use misleading and ambiguous language in their terms and conditions and privacy policies.

There is a lack of transparency in the practice of data brokers, and on the way from the source to the data product, data may change hands many times, and it is challenging to identify all actors in this data value chain.

According to The Vermont Statutes, a first-of-its-kind bill to regulate data brokers went into effect in January 2019. Data brokers, i.e., businesses collecting and selling data about Vermont, USA, residents are required to register and to share information with the public about how they operate. But the Vermont law only covers third-party data companies, while the first-party data holders that collect data directly from users, such as Google, Amazon, or Facebook, are not covered by this law. Despite the big list of firms registered, there is little clear information about what these firms are doing with the data and whether users can remove themselves from their database [83].

On 15 December 2020, the EU released drafts of the long-awaited Digital Services Act and Digital Markets Act, proposing measures to regulate online platforms to protect consumers and competition. The Digital Services Act includes rules for intermediary services (Internet access providers, domain name registrars), hosting services, online platforms, and very large online platforms. The Digital Markets Act includes rules for gatekeeper online platforms aimed at prohibiting unfair practices by them [84]. The gatekeeper may also have a role as device manufacturer and developers of operating systems. Consequently, with all these actors and their multiple roles, the complex IoT ecosystem becomes even more complex.

## 9. Conclusions

There is no single Internet of Things definition as it is still evolving, together with the IoT evolution. A similar situation can be seen in the case of IoT architecture, as there is no standard IoT architecture, while about a thousand IoT related standards are present.

In this regard, IoT complexity and numerous perspectives have led to different IoT models being proposed by many researchers, communities, and organizations. However, to our knowledge, there is a lack of research from a regulatory perspective.

Bearing in mind that the IoT ecosystem involves various IoT actors, regulatory challenges are significantly greater than before. Intending to identify IoT components, IoT actors and relationships among them, we made a comparison of various approaches and mappings between identified IoT actors. We believe that this mapping of IoT actors from various perspective, along with the presented IoT model, gives a clearer picture and better clarification of the blurred IoT actors’ relationships.

IoT devices generate large amounts of data, so data management is one of the biggest challenges in the IoT. As the significance of data and data related activities are increasing, consequently the significance of data laws, regulations, and policies are also increasing. Here, the GDPR is of extreme importance, together with draft ePrivacy Regulations.

Because of the high degree of fragmentation between the many IoT actors, a high risk to data protection exist. In that way, keeping in mind existing complexity in defining controller, joint controller, and processor roles and the distribution of responsibilities among them, our contribution could help relevant authorities to better understand the data management layers. The situation is further complicated if we consider that joint controllers, according to the GDPR, are not obliged to share their responsibilities equally. Now, the real test for the GDPR is in its enforcement, and future challenges lie in clarifying how to apply the GDPR principles to technologies such as IoT, as stated in the European Commission first evaluation and review of GDPR.

Recent research suggests that the future of IoT lies in combining the advantages of multiple computing paradigms. Firstly, Cisco introduced a simplified model of three IoT computation stack and data management layers placed in the edge layer, in the fog layer, and in the cloud layer. Later, NIST presented a conceptual model of fog and mist computing aimed at facilitating meaningful conversations on the topic. Comparing these models, it can be noticed that Cisco’s model does not identify the mist layer, while the NIST recommendations only give focus to fog and mist computing, emphasizing that fog computing is hierarchical, while edge computing is limited to a modest number of peripherals. Furthermore, a four-layer platform has also been evolved, namely the cloud-fog-edge-dew computing model. Compared to the Cisco model, this model introduces dew computing with the primary aim of enabling content when there is no Internet connectivity. Compared to the NIST model, this model does not include mist computing, while it does identify dew computing and edge computing.

Acknowledging that there are other similar computing paradigms and that some of these computing paradigms are a sub-set of others, we present the new five-layer IoT model, where a symbiosis of cloud-fog-edge-mist-dew computing paradigms exists. In this regard, as data controllers and data processors must set up appropriate technical and organizations measures to achieve the data protection principles required by the GDPR, our model is focuses on device processing capabilities and computing paradigms. From that perspective, we posit here the granulation of the Data Manager role in the IoT model in order to better understand where the responsibility for managing the capture, storage, transferring, and processing of IoT data begins. It is evident from our model that all IoT actors have their share of data protection responsibility, from IoT Developer to End user.

In future work, we plan to explore the relationships between the identified IoT actors, data brokers, and large online platforms.

## Figures and Tables

**Table 1:** General Data Protection Regulation (GDPR) actors.

GDPR Actor	Description by the GDPR [9]
Controller	Article 4 point (7) ‘’controller means the natural or legal person, public authority, agency or other body which, alone or jointly with others, determines the purposes and means of the processing of personal data’’. Article 35 paragraph 1. ‘’Where a type of processing in particular using new technologies, and taking into account the nature, scope, context and purposes of the processing, is likely to result in a high risk to the rights and freedoms of natural persons, the controller shall, prior to the processing, carry out an assessment of the impact of the envisaged processing operations on the protection of personal data.’’
Joint Controller	Article paragraph 26 ‘’Where two or more controllers jointly determine the purposes and means of processing, they shall be joint controllers’’.
Processor	Article 4 point (8) ‘’processor means a natural or legal person, public authority, agency, or other body which processes personal data on behalf of the controller’’.
Third Party	Article 4 point (10) ‘’third party means a natural or legal person, public authority, agency or body other than the data subject, controller, processor and persons who, under the direct authority of the controller or processor, are authorised to process personal data’’.
Data Protection Officer (DPO)	Article 37 paragraph 1. ‘’The controller and the processor shall designate a data protection officer in any case where:’’…b) ‘’the core activities of the controller or the processor consist of processing operations which, by virtue of their nature, their scope and/or their purposes, require regular and systematic monitoring of data subjects on a large scale;’’ Article 37 6. ‘’The data protection officer may be a staff member of the controller or processor, or fulfil the tasks on the basis of a service contract’’
Supervisory Authority	Article 51 paragraph 1 ‘’Each Member State shall provide for one or more independent public authorities to be responsible for monitoring the application of this Regulation, …’’
Lead Supervisory Authority	Article 56 paragraph 1 ‘’… the supervisory authority of the main establishment or of the single establishment of the controller or processor shall be competent to act as lead supervisory authority for the cross-border processing carried out by that controller or processor…’’ Article 56 paragraph 6 ‘’The lead supervisory authority shall be the sole interlocutor of the controller or processor for the cross-border processing carried out by that controller or processor. ‘’

**Table 2:** Computing hardware locations and corresponding Internet of Things (IoT) actors.

	Cloud Computing	Fog Computing	Edge Computing	Mist Computing	Dew Computing
Computing hardware location	Large Cloud, Data centres	Mini Cloud smart gateways or routers.	The first hop from the IoT device Wi-Fi access points, switches, or gateways	The far edge of the IoT network It usually has microchips or microcontrollers built into the device	Server located inside the user’s PC and Information processing devices
IoT actor	Cloud Data Manager Cloud Service Provider	Fog Data Manager Network equipment provider Network providers or other business	Edge Data Manager Network equipment provider Network providers or other business	Mist Data Manager Device provider Network providers or other business	Dew Data Manager Device provider Users Self-organized, local, or other business

**Table 3:** Mappings between the IoT actors and business roles in selected International Telecommunication Union (ITU) recommendations.

IoT Actors Identified in ITU Recommendations ITU-T Y.4100 [6] and ITU-T Y.4114 [7]	Business Roles in Informative Appendix I of Recommendation ITU-T Y.4000 [5]
Data Manager is responsible for managing the capture, processing, storage, and transfer of IoT data to meet the IoT service provision requirements [6]. Data manager actor can be a human Data manager or a machine Data manager actor	Application provider Device provider
Service Provider provides services related to things, such as location tracking, monitoring, and service discovery [6].	Application provider, Platform provider, Network provider.
IoT User uses services related to things, such as location tracking, monitoring, and service discovery [6].	Application customer
IoT Data Provider collects data from things and injects the data processed within the IoT system as well as data from external sources and provides them via the IoT data carrier to the IoT data consumer [7].	Device provider, Network provider, Platform provider, Application provider
IoT Data Consumer consumes IoT data. Usage of the consumed data depends on application purposes [7].	Device provider, Network provider, Platform provider, Application provider, Application customer.
IoT Data Framework Provider provides general IoT data processing capabilities and related infrastructure (e.g., storage and computing resources, data processing run time environment) as required by the IoT data provider, IoT data carrier, IoT data application provider, and IoT data consumer for the support of data operations execution [7].	Network provider, Platform provider.
IoT Data Application Provider provides applications related to the execution of IoT data operations (e.g., applications for data analysis, data pre-processing, data visualization, and data query) [7].	Device provider, Network provider, Application provider.
IoT Data Carrier carries data among the IoT data provider, the IoT data framework provider, the IoT data application provider, and the IoT data consumer [7].	Network provider.

**Table 4:** Mappings between the IoT actors identified from various perspectives.

IoT Actors	IoT Actors Identified from Various Perspectives
IoT Developer	IoT service developer [17] IoT application developer [17]
IoT Security Specialist	Security specialists [64]
IoT Data Protection and Privacy Specialist	Data protection officer [6]
IoT Data Manager	Data manager [6] Application provider + data management [5] Device provider + data management [5]
IoT Device Provider	Device provider [5,17,65] Device manufacturers, module manufacturers [66] Designers and producers of connected devices [67] IoT module providers [67] The designers and manufacturers of the objects [64] The manufacturers of the module components [64] Device, component, and chipset manufacturers [68] Device manufacturer/provider [69] Device manufacturers, component manufacturers [70]
IoT Network equipment Provider	Suppliers of the middleware [64] Network equipment providers [67] Infrastructure manufacturers [70] Network equipment manufacturers [64] Connectivity equipment developers and vendors [68]
IoT Platform Provider	Connectivity platforms [39] Device management platforms [39] Cloud platforms [39] Application enablement platforms [39] Advanced analytics platforms [39] IoT platform provider [67], [17] Platform vendors [70] Platform provider [65], [5]
IoT Connectivity Provider	Network provider [5,57,64] Infrastructure provider [17] Operators [65] Connectivity service provider [43] Connectivity provider (network developer) [71] Connectivity provider [17,66,67] Connectivity/network provider [69] Connectivity/mobile network operators [68] Middleware/analytics vendors (connectivity providers, service provider) [70]
IoT Service Provider	Service provider [6,66,69]. (application provider, platform provider, network provider) [5] IoT service provider [43] Service providers and data aggregators [64] Service (cloud service providers, IoT platforms) [68] Service enabler and service creator [71] Cloud computing companies [64], IoT cloud provider [67]
IoT Application Provider	Application provider [5,65,69]
IoT Integrator	IoT service integrator [17] Integrators [64], System integrator [65,66]
IoT User	IoT user [17,43] Application customer [5]
End User	End user [17,43,69] Markets-payers (consumer, end user, company, public sector) [68]

## Data Availability

Data sharing not applicable.
